# A Comprehensive Management of Devic's Disease: A Pediatric Case Study

**DOI:** 10.7759/cureus.62512

**Published:** 2024-06-17

**Authors:** Anandi R Dave, Snehal S Shamal, H V Sharath

**Affiliations:** 1 Department of Neurophysiotherapy, Ravi Nair Physiotherapy College, Datta Meghe Institute of Higher Education & Research, Wardha, IND; 2 Department of Paediatric Physiotherapy, Ravi Nair Physiotherapy College, Datta Meghe Institute of Higher Education & Research, Wardha, IND

**Keywords:** pediatric physiotherapists, devic's disease, pediatric balance scale, pediatric neuromyelitis optica, immunoglobulin therapy, pedi, fatigue severity scale, neurophysiotherapy rehabilitation, neuromyelitis optica spectrum disorder (nmosd)

## Abstract

Devic's disease, also known as neuromyelitis optica (NMO), is an uncommon autoimmune condition that affects the optic nerves and spinal cord. It is characterized by recurrent optic neuritis and myelitis, which can cause paralysis and visual impairment. Because NMO mimics multiple sclerosis, diagnosing it is difficult and necessitates particular testing, such as magnetic resonance imaging (MRI) and aquaporin-4 antibody detection. Patients with NMOs are susceptible to severe, erratic episodes that can result in rapid impairment. As such, timely and efficient therapy with immunosuppressive medicines and continued supportive care are crucial. Improving mobility, strength, coordination, and quality of life while treating the functional deficiencies associated with NMOs requires the use of physiotherapy. This case study emphasizes how crucial it is to manage a young NMO patient using a multidisciplinary strategy in order to maximise results. This case report discusses a 16-year-old male presenting with a sudden onset of balance impairment, slurred speech, difficulty walking and breathing, and weakness in limbs, with the right side more affected. Over three months, he experienced increasing eyesight issues, fatigue, tremors during activities of daily living, difficulty swallowing, and night cramps. Diagnostic investigations including MRI, angiography, visual evoked potentials (VEP) study, and cerebrospinal fluid (CSF) analysis confirmed demyelinating changes consistent with NMO, also known as Devic's disease. The patient received management with steroidal medications, immunosuppressants, and plasma therapy, along with physiotherapy rehabilitation. The physiotherapy protocol aimed to address muscle weakness, coordination impairment, balance issues, fine motor deficits, fatigue, sensory impairment, and dependence on activities of daily living. Motor, sensory, and cranial nerve assessments were conducted, revealing impairments consistent with NMO. Outcome measures pre- and post-intervention showed improvements in functional independence, balance, and fatigue severity. The medical management included a combination of medications and investigations to manage NMO symptoms and monitor disease progression. The physiotherapeutic approach employed a multidisciplinary strategy focusing on education, exercise, and functional tasks to improve the patient's quality of life and independence.

## Introduction

Devic's disease, also known as neuromyelitis optica (NMO), is a rare autoimmune disorder primarily affecting the optic nerves and spinal cord [1,2]. It is characterized by inflammation and demyelination of these central nervous system structures, leading to a range of neurological symptoms [3,4]. It frequently manifests as transverse myelitis, which causes limb weakness, sensory loss, and bladder/bowel problems, as well as optic neuritis, which causes vision loss. The prevalence of NMO is estimated to be around 1-2 individuals per 100,000 in the general population, making it less common than conditions like multiple sclerosis (MS) [5]. The exact cause of NMO is not fully understood, but it is thought to involve an autoimmune response targeting a protein called aquaporin-4, which is found in the optic nerves and spinal cord [6,7].

Diagnostic investigations for NMO include magnetic resonance imaging (MRI) scans, which may reveal characteristic features such as myelitis, and blood tests to detect specific antibodies like aquaporin-4 [8]. Lumbar puncture (cerebrospinal fluid (CSF) analysis) may also be conducted to assess for abnormalities indicative of NMO [9,10]. Signs and symptoms of NMO can vary but often include optic neuritis, leading to visual disturbances, and transverse myelitis, resulting in limb weakness, numbness, and loss of bladder and bowel control [11,12]. Other symptoms may include slurred speech, balance difficulties, and pain. NMO typically follows a relapsing-remitting course, with periods of exacerbation and partial or complete recovery [13,14]. Corticosteroids, plasma exchange for acute episodes, and immunosuppressive medication for long-term care are all included in the treatment plan. Enhancing mobility, strength, coordination, and general quality of life with physiotherapy necessitates a multidisciplinary approach for the best possible patient treatment.

Physiotherapy plays a crucial role in managing NMO by addressing functional deficits and enhancing the patient's overall quality of life [15,16]. Physiotherapeutic interventions focus on improving mobility, balance, and coordination [17]. Specific exercises are designed to address muscle weakness, spasticity, and impaired gait. Moreover, physiotherapists employ neurorehabilitation techniques to enhance motor control and re-educate neural pathways [18,19]. This comprehensive approach helps individuals with NMO regain independence in daily activities, minimize the impact of symptoms, and prevent complications associated with immobility. Regular physiotherapy sessions contribute to a holistic management strategy for individuals living with NMO.

## Case presentation

We are discussing a case report representing a 16-year-old male who presented in October 2023 with complaints of sudden balance impairment, slurred speech, and difficulty in walking and breathing. He reported weakness in his limbs, with the right side being more affected. Additional history included a recent increase in myopia, fatigue, the occurrence of tremors during activities of daily living (ADLs), difficulty swallowing, and night cramps. All these symptoms manifested over the span of three months.

Upon admission to the neurology department, the following investigations were conducted: MRI, angiography, a visual evoked potentials (VEP) study, and CSF analysis. These investigations revealed demyelinating changes. The diagnosis was confirmed through NMO and myelin oligodendrocyte glycoprotein (MOG) antibody tests. The patient was managed with steroidal medications, immunosuppressants, and plasma therapy.

Simultaneously, a physiotherapy rehabilitation program was initiated. A thorough assessment was conducted, and a physiotherapy protocol was planned accordingly. The assessments included evaluations of reflexes, muscle strength (manual muscle testing (MMT)), cranial nerve function, and sensory function. The detailed results are presented in the tables below.

Motor assessment

Prior to conducting reflex examinations and MMT, the patient's consent was obtained (Table 1 and Table 2).

**Table 1:** Reflexes Ab: absent; +: reflex diminished; ++: normal reflex

Reflexes	Right	Left
Biceps reflex	++	++
Triceps reflex	++	++
Brachioradialis reflex	+	+
Patellar reflex	Ab	Ab
Achilles reflex	Ab	Ab

**Table 2:** MMT 1: flickering contraction; 2: full range of motion in gravity eliminated plane; MMT: manual muscle testing

Joints	MMT grade (right)	MMT grade (left)
Shoulder flexors	1	2
Shoulder extensors	1	2
Elbow flexors	1	2
Elbow extensors	1	2
Wrist flexors	1	2
Wrist extensors	1	2
Hip flexors	2	2
Hip extensors	2	2
Knee flexors	2	2
Knee extensors	2	2
Ankle plantar flexors	2	2
Ankle dorsiflexors	2	2

Cranial nerve assessment 

A thorough cranial nerve examination was done which revealed impairment of the optic nerve. All other cranial nerves are intact.

Sensory assessment

Patient consent was obtained prior to assessing sensations, as indicated in Table 3.

**Table 3:** Sensory assessment

Sensation	Upper limbs		Lower limbs	
Superficial	Right	Left	Right	Left
Pain	Diminished	Diminished	Diminished	Diminished
Light touch	Diminished	Diminished	Diminished	Diminished
Temperature	Diminished	Diminished	Diminished	Diminished
Deep	Right	Left	Right	Left
Vibration	Intact	Intact	Intact	Intact
Kinesthesia	Intact	Intact	Intact	Intact
Proprioception	Intact	Intact	Intact	Intact
Cortical	Right	Left	Right	Left
Two-point discrimination	Intact	Intact	Intact	Intact
Stereognosis	Intact	Intact	Intact	Intact
Graphesthesia	Intact	Intact	Intact	Intact

Timeline of events 

The patient underwent different investigations, and the timeline is mentioned in Table 4, to simplify the occurrence of events.

**Table 4:** Timeline of events MRI: magnetic resonance imaging; CSF: cerebrospinal fluid; VEP: visual evoked potentials

Timeline	Events
23 Oct 2023	Got admitted
24 Oct 2023	Investigations were done (MRI of the brain and spine and CSF study)
25 Oct 2023	Treatment with medications
27 Oct 2023	VEP study
29 Oct 2023	Plasma therapy started
30 Oct 2023	Physiotherapy intervention started
20 Dec 2023	Latest date of outcome assessment

Investigation and medical management

The investigations the patient went through are mentioned with the drugs given on the day of screening in Table 5.

**Table 5:** Investigation and medical management Tab: tablet; mg: milligram; MRI: magnetic resonance imaging; NMOSD: neuromyelitis optica spectrum disorder; LFT: liver function test; CNS: central nervous system; IgG: immunoglobulin G; MOG: myelin oligodendrocyte glycoprotein; USG: ultrasonography; CE-MRI: contrast-enhanced magnetic resonance imaging; NMO: neuromyelitis optica; VEP: visual evoked potentials; CSF: cerebrospinal fluid

Date	Medical management	Investigation
23 Oct 2023	Tab. Mepresso T (8mg); Tab. Pan (40mg); Tab. Diamox (250mg); Tab. Dynapar MR; Tab. Ivabrad (5mg); Tab. Met XL (25mg); Tab. Nicardia R (20mg)	Cervical MRI reveals features of myelitis from C1 to D3; brain MRI and angiography revealed brainstem myelitis confirming NMOSD; LFT, serology blood glucose, and coagulation protein were normal
25 Oct 2023	Tab. Mepresso T (8mg); Tab. Pan (40mg); Tab. Diamox (250mg); Tab. Dynapar MR; Tab. Ivabrad (5mg); Tab. Met XL (25mg); Tab. Nicardia R (20mg)	CSF study in oligoclonal bands shows intrathecal synthesis in CNS and shows the presence of an IgG band in the immunofixation pattern of CSF, and antibodies against MOG antibodies serum were seen
27 Oct 2023	Inj. Targocid (400mg); Inj. Optineuron (1amp); Inj. Pantop (40mg); Inj. Vitamin K (10mg)	VEP study shows delayed P100 latencies in both eyes which is suggestive of bilateral anterior visual pathway abnormality (demyelination)
2 Dec 2023	Inj. Targocid (400mg); Inj. Meropenem (500mg); Inj. Optineuron (1 A in 100ml NS IV0 Inj. Pan (40mg) Inj. Emset (4mg); Tab. Gabapentin (100mg)	USG of the abdomen and pelvis showed altered echotexture of the liver which reveals cystitis
10 Dec 2023	Tab. Gabapentin; Tab. Pan D Immunoglobulin	Spine MRI with contrast and brain CE-MRI were done which revealed NMO from C3 to D3 and disc degeneration from L5 to S1 level

Physiotherapeutic management

A comprehensive physiotherapy protocol was developed based on the initial assessment, focusing on muscle strengthening, coordination, balance, fine motor skills, and endurance. Detailed intervention is mentioned in Table 6.

**Table 6:** Physiotherapeutic management ROM: range of motion; ADLs: activities of daily living

Sr. no.	Problem list	Goal	Intervention	Dosage	Rationale
1	Patient education	Improve understanding of the condition and rehabilitation process	Provide information on the condition, treatment plan, and importance of adherence to the rehabilitation program to the patient and parents	Throughout sessions	Enhances patient compliance and active participation in rehabilitation program
2	Muscle weakness	Improve muscle strength and endurance	Strengthening exercises: active ROM exercises for bilateral upper and lower limbs, progressing with weights	3 times a week, 10 repetitions × 1 set	Increases muscle strength and endurance, facilitating functional activities and mobility
3	Coordination impairment	Enhance coordination and balance	Functional task-oriented approach	Throughout sessions	Improves coordination and motor control necessary for daily activities and gait
4	Balance impairment	Improve balance and stability	Gait training: sit-to-stand, progressing to squats, spot marching, obstacle walking, wobble board with support	3 times a week	Enhances balance, reduces risk of falls, and improves mobility
5	Fine motor impairment	Enhance fine motor skills	Task-oriented approach	Throughout sessions	Improves skills essential for activities like writing, dressing, and self-care
6	Fatigue	Improve endurance	Cardiovascular endurance exercises: diaphragmatic exercises, incentive spirometry, Jacobson's relaxation technique	3 times a week, as tolerated	Enhances endurance, reduces fatigue, and improves overall stamina and tolerance
7	Superficial senses impaired	Enhance sensory awareness	Sensory re-education exercises	Throughout sessions	Improves sensory awareness and perception
8	Dependent to perform ADLs	Increase independence in ADLs	Functional task-oriented approach for ADLs	Throughout sessions	Promotes independence and confidence in performing daily activities and improves quality of life

Outcome measures

The outcome measures were evaluated at the beginning and at the end of the physiotherapy treatment period as mentioned in Table 7.

**Table 7:** Outcome measures taken pre- and post-intervention FSS: Fatigue Severity Scale; FIM: Functional Independence Measure; PEDI: Pediatric Evaluation of Disability Inventory; PBS: Pediatric Balance Scale

Outcome measures	Pre-intervention	Post-intervention
FSS	59	33
FIM	89	104
PEDI	96	74
PBS	28	41

## Discussion

A 16-year-old male patient with NMO is described in this case study. He presented with sudden balance impairment, slurred speech, breathing and walking difficulties, limb weakness (particularly on the right side), vision issues, exhaustion, tremors, difficulty swallowing, and night cramps. His symptoms worsened over the course of three months, leading to a hospital stay. There, diagnostic tests such as an MRI, a VEP study, and a CSF analysis showed NMO. In addition to receiving immunosuppressants, steroids, and plasma therapy for medical care, the patient also participated in an organised physical therapy rehabilitation program.

Nechemia et al. conducted a study focusing on the efficacy of multidisciplinary inpatient rehabilitation for individuals diagnosed with NMO, comparing their outcomes with patients diagnosed with MS. The objective was to evaluate the extent to which existing rehabilitation protocols designed for MS could be adapted and effective for NMO patients [20]. The study analyzed data from 15 NMO and 32 MS inpatients, assessing various parameters to gauge the effectiveness of rehabilitation interventions. One notable finding was the longer length of stay required for NMO patients compared to those with MS. Despite this difference, both groups experienced significant benefits from the rehabilitation program. Specifically, NMO patients demonstrated greater improvements in Functional Independence Measure (FIM) scores compared to MS patients. The FIM scores serve as a comprehensive measure of a patient's ability to perform ADLs and their level of independence. The higher improvement in FIM scores among NMO patients suggests that the rehabilitation interventions were particularly effective in addressing the functional deficits associated with NMO [20].

Furthermore, the study observed lower scores on the Expanded Disability Status Scale (EDSS) at discharge for NMO patients compared to MS patients. The EDSS is a widely used scale to quantify disability in MS, assessing various functional systems affected by the disease. The lower EDSS scores in NMO patients indicate less disability and better functional outcomes following the rehabilitation program. These findings underscore the potential for significant functional gains in NMO patients through tailored rehabilitation interventions. Despite the inherent challenges and complexities associated with NMO, such as optic nerve involvement and spinal cord inflammation, the study suggests that rehabilitation strategies developed for MS can be adapted and effectively applied to NMO with appropriate modifications. The study by Nechemia et al. emphasizes the importance of individualized rehabilitation approaches that address the specific needs and challenges of NMO patients. By tailoring interventions to target the unique symptoms and impairments associated with NMO, healthcare providers can optimize outcomes and enhance the quality of life for individuals living with this rare autoimmune disorder.

## Conclusions

This case report highlights the importance of a comprehensive multidisciplinary approach in managing NMO. Early diagnosis and prompt initiation of medical management, including immunosuppressive therapy, are crucial in controlling disease activity and preventing relapses. Physiotherapy plays a vital role in addressing functional deficits and enhancing the patient's overall quality of life. Tailored physiotherapeutic interventions focusing on muscle strengthening, coordination, balance, fine motor skills, endurance, and sensory awareness contribute to improving mobility and independence in ADLs and reducing the impact of symptoms such as fatigue. Regular monitoring of outcomes and adjustment of interventions based on the patient's progress are essential for optimizing rehabilitation outcomes in individuals with NMO.

The 16-year-old male patient with NMO showed significant improvements following a structured four-week physiotherapy rehabilitation program. Initially presenting with severe balance impairment, slurred speech, limb weakness, vision problems, fatigue, tremors, and difficulties in walking, breathing, and performing ADLs, the patient underwent a comprehensive intervention plan targeting these impairments. Post-intervention assessments revealed notable enhancements in muscle strength, coordination, balance, and overall mobility. The FIM score increased, indicating greater functional independence, while the Pediatric Balance Scale (PBS) score improved, reflecting better balance and stability. The patient's endurance and fine motor skills also saw significant progress, leading to increased independence in ADLs and an overall improvement in quality of life. This case underscores the effectiveness of tailored physiotherapy interventions in managing NMO, demonstrating that a multidisciplinary and individualized approach can yield substantial functional gains and enhance patient outcomes.
